# Regeneration of *Solanum nigrum* by Somatic Embryogenesis, Involving Frog Egg-Like Body, a Novel Structure

**DOI:** 10.1371/journal.pone.0098672

**Published:** 2014-06-04

**Authors:** Kedong Xu, Yunxia Chang, Kun Liu, Feige Wang, Zhongyuan Liu, Ting Zhang, Tong Li, Yi Zhang, Fuli Zhang, Ju Zhang, Yan Wang, Wei Niu, Shuzhao Jia, Hengchang Xie, Guangxuan Tan, Chengwei Li

**Affiliations:** 1 Key Laboratory of Plant Genetics and Molecular Breeding, Zhoukou Normal University, Zhoukou, People's Republic of China; 2 College of Life Science and Agronomy, Zhoukou Normal University, Zhoukou, People's Republic of China; 3 College of Life Science, Henan Agricultural University, Zhengzhou, People's Republic of China; Instituto Butantan, Brazil

## Abstract

A new protocol was established for the regeneration of *Solanum nigrum* by frog egg-like bodies (FELBs), which are novel somatic embryogenesis (SE) structures induced from the root, stem, and leaf explants. The root, stem, and leaf explants (93.33%, 85.10%, and 100.00%, respectively) were induced to form special embryonic calli on Murashige and Skoog (MS) medium containing 1.0 mg/L 2,4-dichlorophenoxyacetic acid, under dark condition. Further, special embryonic calli from the root, stem, and leaf explants (86.97%, 83.30%, and 99.47%, respectively) were developed into FELBs. Plantlets of FELBs from the three explants were induced *in vitro* on MS medium supplemented with 5.0 mg/L 6-benzylaminopurine and 0.1 mg/L gibberellic acid, and 100.00% plantlet induction rates were noted. However, plantlet induction *in vivo* on MS medium supplemented with 20 mg/L thidiazuron showed rates of 38.63%, 15.63%, and 61.30% for the root, stem, and leaf explants, respectively, which were lower than those of the *in vitro* culture. Morphological and histological analyses of FELBs at different development stages revealed that they are a novel type of SE structure that developed from the mesophyll (leaf) or cortex (stem and root) cells of *S*. *nigrum*.

## Introduction

*Solanum nigrum* is a solanaceous medicinal herb commonly known as “black nightshade.” The plant has been extensively used as a traditional medicine in Asia because it contains valuable medicinal components, including glycoalkaloids (solanine, solamargine, solanigrine, and solasodine) [1], steroidal glycosides (β-solamargine, solasonine, and α, β-solansodamine) [2], steroidal saponins (diosgenin) [3], steroidal genin (gitogenin) [4], and tannin and polyphenolic compounds [5]. The components can help prevent and cure liver disease [6], urinary tract infection [7], and leucorrhea [8] and promote heat clearing, detoxification [9], and dissolving stasis [10]. *S*. *nigrum* extracts exhibit remarkable antimicrobial activity against *Staphylococcus aureus*, *Typhoid bacillus*, *Bacillus cereus*, *Micrococcus kristinae*, *Pseudomonas aeruginosa* [11], and *Shigella dysenteriae* [12]. In addition, its fruit is reported to have antiulcer, antioxidant, and antitumor effects in rats [13]–[15].

Besides its valuable medicinal components, studies on the components of its amino acids, polysaccharides, and active ingredients [16], the mechanisms of cadmium accumulation [17], somatic hybridization [18], [19], anticancer mechanisms [20], herbicidal activity [21], fruit pigment [22], karyotype analysis [23], protoplast isolation [24], insecticide and virus resistance [25], [26], endophytic bacteria [27], and antibacterial and antiviral abilities [28], [29], have also been conducted. However, high-frequency regeneration and transformation systems in *S*. *nigrum* have not been established, even though low-frequency protoplast transformation with *Agrobacterium rhizogenes* [30], and direct regeneration [31], has been reported. Somatic embryogenesis (SE) offers a regeneration system with the following advantages: high propagation rates, labor saving, suitable for suspension cultures, and plantlets can be produced from genetically modified single cells. Regeneration by SE has often been used in germplasm preservation and in establishing high-efficiency transformation systems. To our knowledge, no study on SE in *S*. *nigrum* has been conducted. In this study, a regeneration system in *S*. *nigrum* was established through frog egg-like bodies (FELBs), novel SE structures, which may be used in future studies of *S*. *nigrum*.

## Materials and Methods

### Plant materials and explant preparation

*S*. *nigrum* seeds were treated with 75% (v/v) ethanol for 30 s, rinsed with sterilized distilled water three times, soaked in 2.5% (v/v) sodium hypochlorite for 8–10 min, and rinsed with sterilized distilled water five times. For germination, the sterilized seeds were sown on Murashige and Skoog (MS) medium [32] supplemented with 0.1 mg/L gibberellic acid (GA_3_), 30 mg/L sucrose, and 7.8 g/L agar (pH 5.8), at 4°C for 4 d, then incubated in a germination chamber (25°C in the dark) until the seeds were fully germinated. Seedlings were transplanted onto MS medium at 25°C with 16 h light (120 µmol·m^−2^s^−1^) and 8 h dark.

### Induction of FELBs

**Figure 1:**
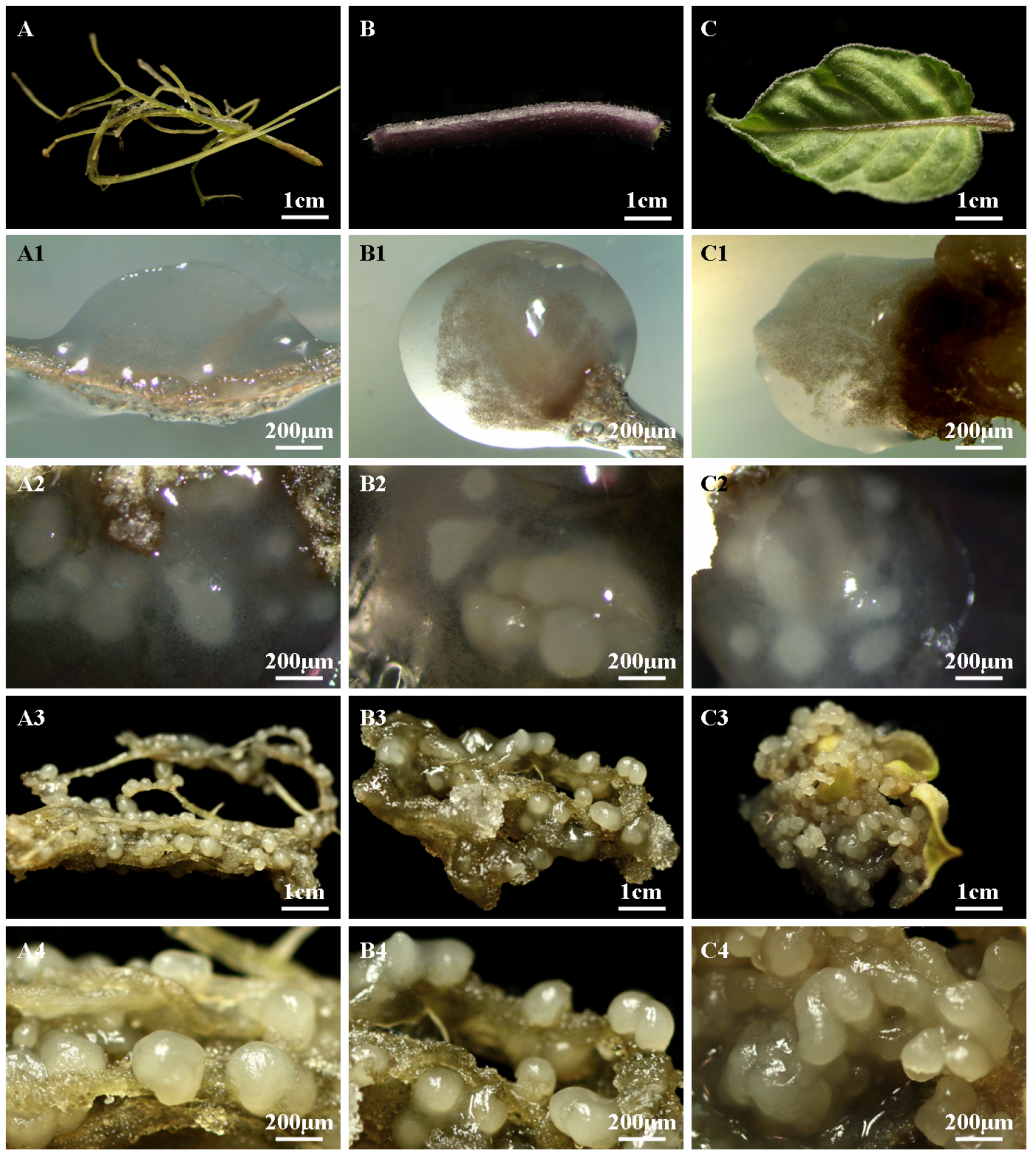
The development of sticky callus and frog egg-like bodies (FELBs) from the leaf, root, and stem explants of *S*. *nigrum*. A, B and C represent root, stem and leaf explants, respectively; A1, B1 and C1 represent translucent sticky callus induced from root, stem and leaf explants, respectively; A2, B2 and C2 represent FELBs at an early stage of development induced from root, stem and leaf explants, respectively; A3, B3 and C3 represent FELBs at a late stage of development induced from root, stem and leaf explants, respectively; A4, B4 and C4 represent enlarged views of FELBs from parts of A3, B3 and C3.

For the optimization of supplementary plant growth regulators, the leaf, root, and stem explants were placed on MS media with 30 g/L sucrose and 7.8 g/L agar, pH 5.8, supplemented with 2,4-dichlorophenoxyacetic acid (2,4-D) or 1-naphthaleneacetic acid (NAA) with the concentration series 0, 0.5, 1.0, and 1.5 mg/L. The explants were kept at 25±1°C and in the dark to induce FELBs. The different stages of SE were recorded by using a digital single lens reflex camera (EOS 600D, Canon Inc., Japan) (Fig. 1 A, A3 and A4; B, B3 and B4; C, C3 and C4) and a stereomicroscope (SMZ800, Nikon Corporation, Japan) (Fig. 1 A1 and A2; B1 and B2; C1 and C2).

### Histochemical and histological analyses of the origin and development of FELBs

**Figure 2:**
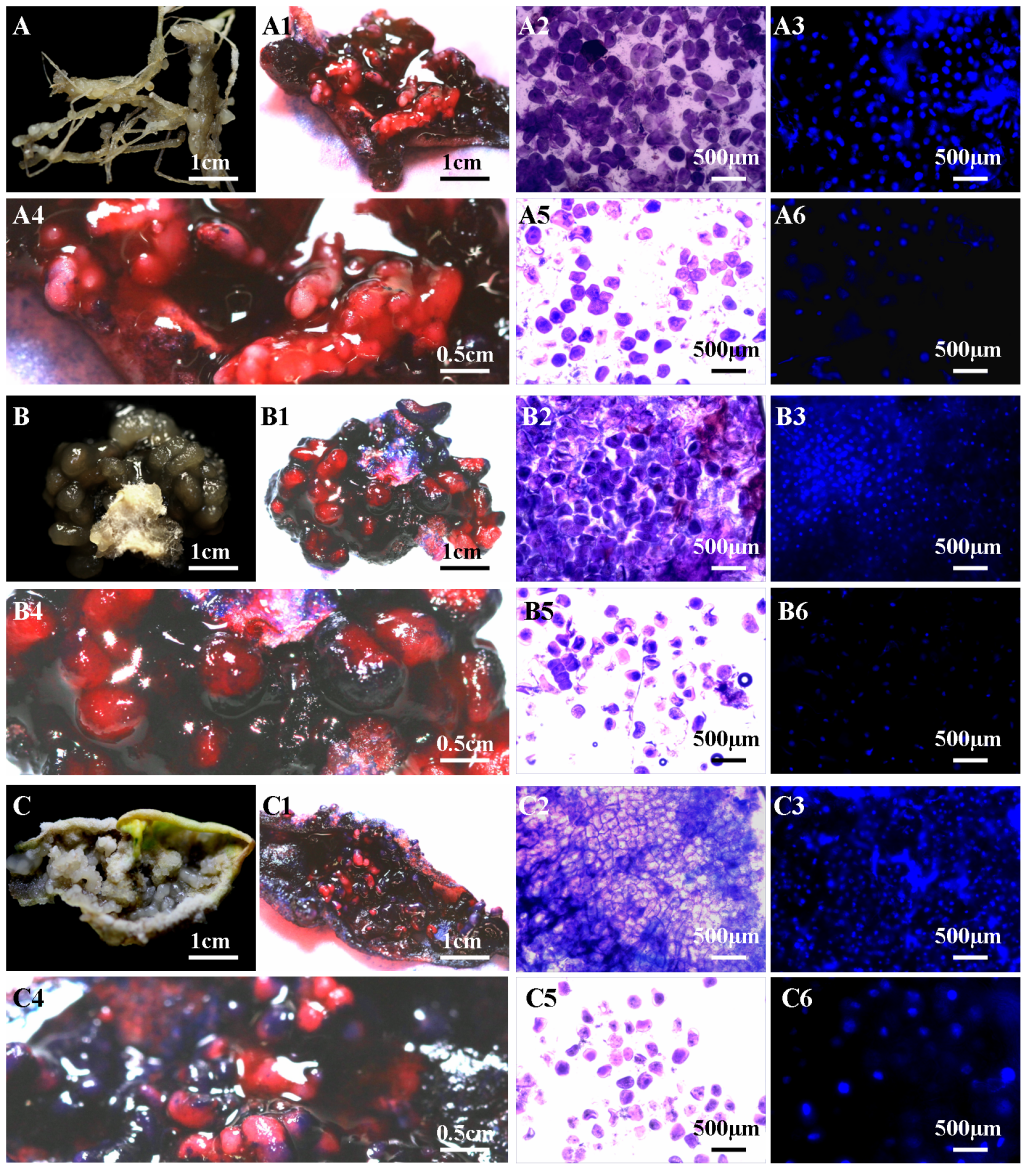
Identification and morphological analysis of embryoids and the non-embryogenic callus of frog egg-like bodies (FELBs). A, B and C represent root, stem and leaf explants, respectively; A1, B1 and C1 represent FELB identification after double staining with acetocarmine and Evans blue for FELB induced from root, stem and leaf explants, respectively; A2, B2 and C2 represent cell morphology and arrangement of FELB embryoids stained with borax-toluidine blue for FELB embryoids induced from root, stem and leaf explants, respectively; A3, B3 and C3 represent cell nuclei of FELB embryoids stained with DAPI and observed in dark-field lighting for FELB embryoids induced from root, stem and leaf explants, respectively; A4, B4 and C4represent enlarged view of A2, B2 and C2, respectively, showing FELB morphology; A5, B5 and C5 represent cell morphology and arrangement of non-embryogenic callus of FELBs stained with borax-toluidine blue, for FELBs induced from root, stem and leaf explants, respectively; A6, B6 and C6 represent cell nuclei of non-embryogenic callus of FELBs stained with DAPI and observed in dark-field lighting, for FELBs induced from root, stem and leaf explants, respectively.

For the confirmation of the presence of FELBs, double staining with acetocarmine and Evans blue [33] was used to distinguish embryonic tissue from callus. FELB embryogenic cells were stained bright red, and non-embryogenic callus was stained dark blue. The different SE stages were recorded using a Canon 600 D camera (Fig. 2 A, A1 and A4; B, B1 and B4; C, C1 and C4) or monitored using a digital fluorescence microscope (BX 61, Olympus Corporation, Japan) (Fig. 2 A2, A3, A5, and A6; B2, B3, B5, and B6; C2, C3, C5, and C6).

Staining with 4′,6-diamidino-2-phenylindole (DAPI) was used to detect the nuclei of embryonic and callus cells, following a previously published method [34]. A single layer of cells was placed on a slide, and then photographed with dark-field illumination using an Olympus BX 61 microscope (Fig. 2 A3 and A6; B3 and B6; C3 and C6). Cell outlines were observed after using borax-toluidine blue staining; fresh material was put in borax-toluidine blue for 5 min, rinsed with distilled water five times to clean the color off, and the moisture was removed with clean filter paper. Microscopic images were recorded under light-field conditions using an optical microscope (BX 41, Olympus Corporation, Japan) (Fig. 2 A2 and A5; B2 and B5; C2 and C5).

**Figure 3:**
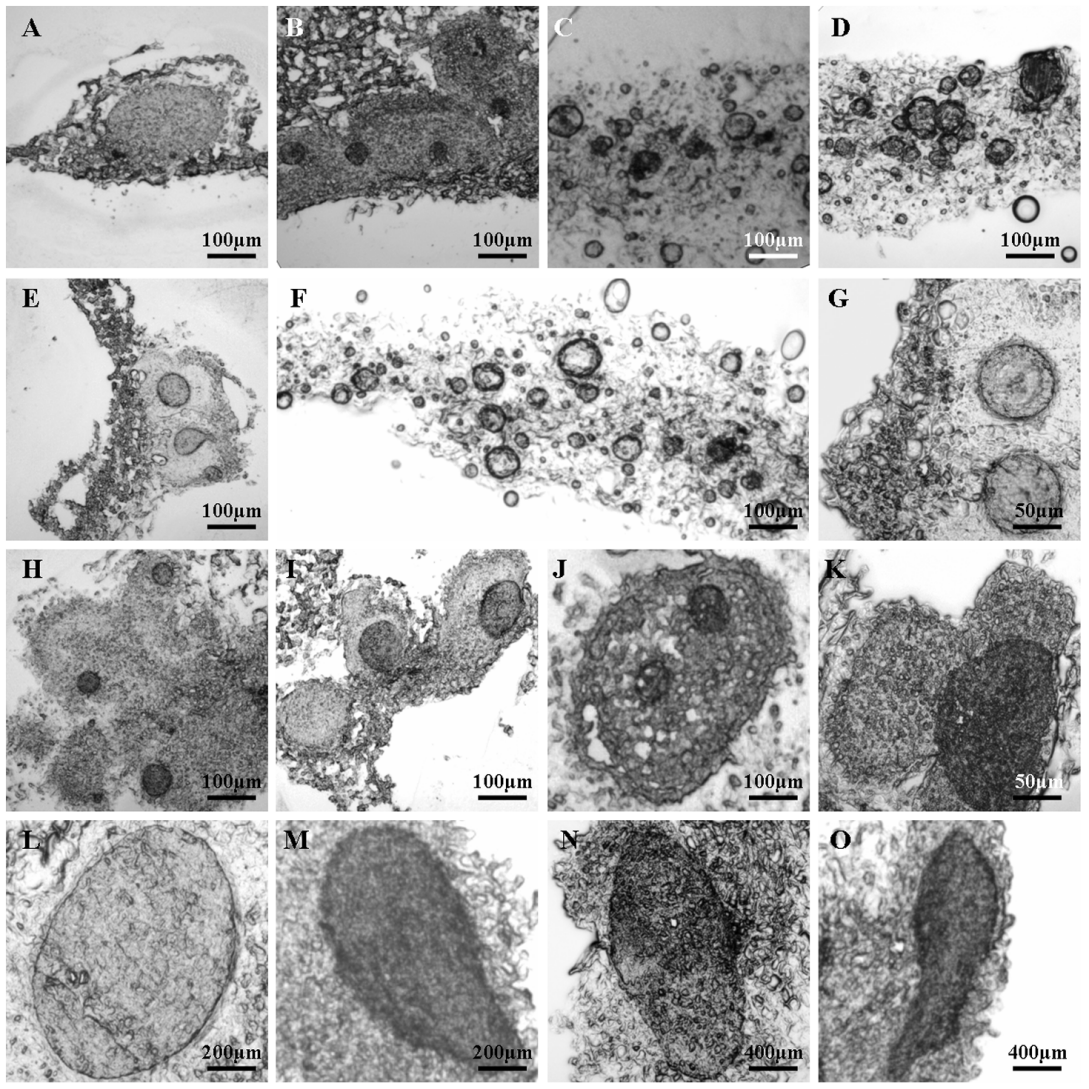
Microscopic images of different stages of SE by frog egg-like bodies (FELBs) in *S. nigrum* by using the frozen section technique. A. Translucent sticky callus induced from a leaf explant; B. FELBs induced from a root explant; C. FELBs induced from a stem explant; D. FELBs induced from a leaf explant; E. The specific localization of FELBs on a leaf explant; F. FELBs containing many embryoids at different developmental stages; G. A spherical, early globular embryo; H-K. Individual FELBs and their embryoids were arranged in different ways, including embryoids in relatively independent FELBs (H and I), two embryoids in one FELB (J), and three embryoids clustered together (K); L-O. Somatic embryos at different developmental stages, including globular shaped embryos (L), heart/torpedo- shaped embryos (M and N), and torpedo- shaped embryos (O).

**Figure 4:**
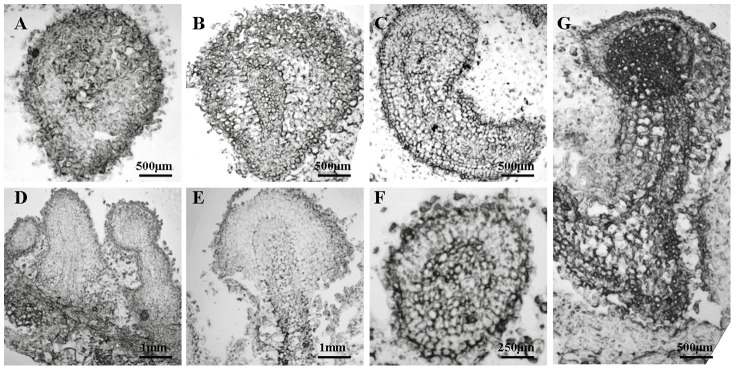
Microscopic images showing the development of vascular tissue in the induced embryoids of frog egg-like bodies (FELBs) and somatic embryos of *S. nigrum*. A. FELB embryoid without vascular tissue forming; B. The appearance of vascular tissue in the center of a FELB embryoid; C. Elongated FELB embryoid with developing vascular tissue; D. Vascular tissue in FELB embryoids at different developmental stages in one view; E. Longitudinal section of developed vascular tissue in a somatic embryo; F. Transverse section of a somatic embryo with developed vascular tissue; G. Developed vascular tissue in a mature somatic embryo.

**Figure 5:**
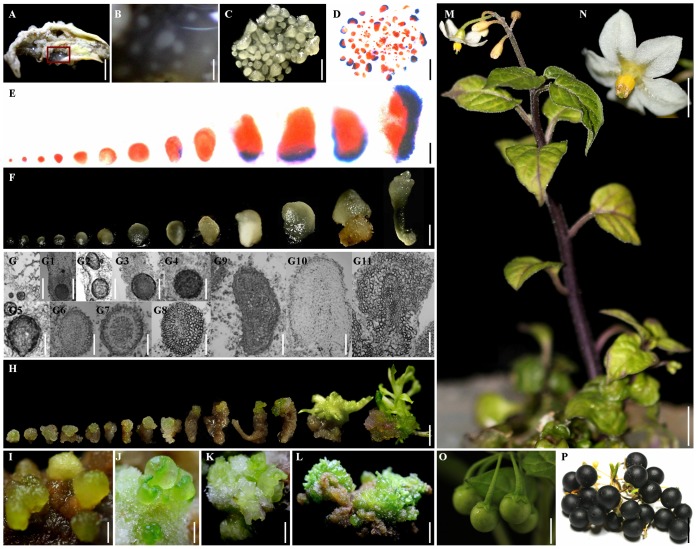
The regeneration process of *S*. *nigrum* by frog egg-like bodies (FELBs). A. Calli induced on a leaf explant; B. Enlarged view of red squares in A, showing FELBs induced in translucent sticky calli; C. Isolated FELBs; D. Isolated FELBs stained with acetocarmine and Evans blue; E. Morphologies of isolated FELBs at different developmental stages stained with acetocarmine and Evans blue; F. Morphologies of the intact individual FELBs at different developmental stages; G. The histological and morphological developmental process of FELBs; H. The regeneration process of FELBs *in vitro*; I, J, K, and L. The regeneration process of FELBs *in vivo*; M. A regenerated seedling; N. A flower from a regenerated plant; O. Immature fruits from a regenerated plant; P. Mature *S*. *nigrum* fruits from a regenerated plant.

The microscopic frozen section method developed by Y. Song (School of Life Science and Technology, Tongji University) was as follows: the materials from the different developmental stages were fixed in 4% paraformaldehyde (dissolved with 100 mM phosphate buffer solution [PBS], pH 7.2) for 48 h, dehydrated in 10% and 20% sucrose for 24 h each, and then embedded in optimum cutting temperature compound (OCT) for 2 h. The embedded tissues were then sectioned to a thickness of 8 µm with a cryostat microtome (CM1850, Leica Microsystems, Germany), and observed under an Olympus BX 41 microscope (Figs. 3, 4, and 5).

### Plantlet formation from *in vitro* and *in vivo* FELBs

Two approaches were used for plantlet formation from FELBs. For the first approach, *in vitro* FELBs were placed on MS medium (pH 5.8) supplemented with 5.0 mg/L 6-benzylaminopurine (BAP) and 0.1 mg/L GA_3_. For the second approach, *in vivo* FELBs in the callus were induced into plantlets by adding different concentrations of thidiazuron (TDZ) (0, 10, 20, and 30 mg/L) in MS medium (pH 5.8). Both *in vitro* and *in vivo* FELBs were cultivated at 25°C under a 16 h photoperiod with light intensity of 120 µmol·m^−2^s^−1^ provided by cool-white fluorescent lights, and were subcultured monthly. Plantlets grown to 1–2 cm were separated and transferred to the rooting medium for root formation. To evaluate the frequency of regenerated plantlets from FELBs, thirty replicates of every 10 FELBs cultivated on 25 mL of medium in 90 mm petri dishes were used.

### Microscopic investigation

Images of cell nuclei were taken using a digital fluorescence microscope (BX 61, Olympus Corporation, Japan) with a mirror unit (U-MNU2), a dichroic mirror (DM400), an excitation filter (BP360), and a barrier filter (BA420) for DAPI [34].

### Statistical analysis

The frequencies of callus and FELB formations influenced by different plant growth regulators were analyzed using analysis of variance (ANOVA), with 99% and 95% confidence intervals, in SPSS 10.0.

## Results

### FELB, a novel SE structure, was induced from the leaf, root, and stem explants of *S*. *nigrum*

Ten days after the inoculation of the explants, obvious, translucent sticky calli (Fig. 1 A1, B1, and C1) had appeared on all three types of explant. Fifteen days after the appearance of the special calli, small white embryoid bodies had formed in the calli (Fig. 1 A2, B2, and C2). At the late developmental stage, FELBs developed into many different individual somatic embryoids, surrounded by less translucent sticky callus (Fig. 1 A3 and A4; B3 and B4; C3 and C4). In most cases, one FELB resulted in more than 10 individual somatic embryoids; sometimes one FELB could give rise to more than 40 somatic embryoids. The SE structure of the special embryogenic callus containing the somatic embryoids looked and functioned like a cluster of frog eggs, inside which the embryoids are protected and receive nutrients from the surrounding callus. Therefore, the entire SE structure was called “FELB”. To our knowledge, our study is the first to show FELBs as novel SE structures in plants.

### Effects of auxin analogs on the induction of translucent sticky calli and FELBs

Two auxin analogs, NAA and 2,4-D, with a concentration series of 0, 0.5, 1.0, and 1.5 mg/L, were used to optimize plant growth regulator conditions for the induction of the translucent calli and FELBs. For all the NAA supplements with their different concentrations, no callus was induced, indicating that NAA would not be suitable for SE in *S*. *nigrum*. For the 2,4-D supplements, a concentration of 1.0 mg/L resulted in significantly higher rates of callus induction in the root, stem, and leaf explants than the other concentrations (93.33%, 85.10%, and 100.00%, respectively; Table 1). Without adding 2,4-D in media, no callus was induced (Table 1), suggesting that adding a suitable plant growth regulator is necessary for callus induction. For a 2,4-D concentration series of 0.5, 1.0, and 1.5 mg/L, the rates of callus induction from different explants exhibited the following sequence: leaf > root > stem.

**Table 1:** Effect of 2,4-D on the induction of translucent sticky callus in the *S*. *nigrum* root, stem, and leaf explants.

2,4-D (mg/L)	Rate (%) of callus induction		
	Root	Stem	Leaf
0	0.00±0.00 jI	0.00±0.00 jI	0.00±0.00 jI
0.5	35.60±0.55 eE	21.73±0.54 gG	57.20±0.50 dD
1.0	93.33±0.54 bB	85.10±0.36 cC	100.00±0.00 aA
1.5	13.47±0.35 iH	20.63±0.59 hG	25.77±0.33 fF

Note: Callus induction rate refers to the ratio of explants with an induced callus to the total number of explants inoculated. The mean and standard error per treatment were calculated from 300 explants from 30 petri dishes (as 30 replicates). Capital and lowercase letters represent significant differences at the 1% and 5% probability levels, respectively. Significant differences were analyzed by the Duncan test, using SPSS 10.0.

Furthermore, we calculated the frequencies of FELB induction from the root, stem, and leaf explants; the frequencies of FELB induction were similar to the rate of callus induction (Table 2), indicating that most translucent sticky calli could develop into FELBs. Supplementing 2,4-D at a concentration of 1.0 mg/L resulted in significantly higher frequencies of FELB induction in the root, stem, and leaf explants than the other concentrations (86.97%, 83.30%, and 99.47%, respectively; Table 2).

**Table 2:** Effect of 2,4-D on frog egg-like body (FELB) induction in the *S*. *nigrum* root, stem, and leaf explants.

2,4-D (mg/L)	Frequency of FELB induction (%)		
	Root	Stem	Leaf
0	0.00±0.00 jJ	0.00±0.00 jJ	0.00±0.00 jJ
0.5	35.13±0.50 eE	17.43±0.34 gG	56.90±0.42 dD
1.0	86.97±0.51 bB	83.30±0.71 cC	99.47±0.25 aA
1.5	12.93±0.29 iI	15.83±0.31 hH	23.87±0.35 fF

Note: Frequency of FELB induction refers to the ratio of explants with induced FELBs to the total number of explants inoculated. The mean and standard error per treatment were calculated from 300 explants from 30 petri dishes (as 30 replicates). Capital and lowercase letters represent significant differences at the 1% and 5% probability levels, respectively. Significant differences were analyzed by the Duncan test, using SPSS 10.0.

### Identification and morphological analysis of FELBs

The embryogenic and callus parts of FELBs were analyzed using double staining with acetocarmine and Evans blue. The embryogenic part, in which the cells reproduce and metabolize quickly, were easily stained bright red, whereas the callus part, in which the cells could not be stained with acetocarmine because of their lower metabolism and reproduction, were detected using Evans blue (Fig. 2 A1 and A4; B1 and B4; C1 and C4). This confirmed that FELBs are composed of red embryoids, surrounded by dark blue translucent sticky calli. We suggest that a FELB is a special SE structure during the early developmental stage of SE in *S*. *nigrum*.

The FELB embryoids were in shape similar to small red balls (Fig. 2 A1 and A4; B1 and B4; C1 and C4). By using microscopic squash technology, together with DAPI staining (Fig. 2 A3 and A6; B3 and B6; C3 and C6) and borax-toluidine blue staining (Fig. 2 A2 and A5; B2 and B5; C2 and C5), embryogenic cells of FELBs were observed to be closely arranged and with thick cytoplasm, while callus cells were much looser in arrangement than embryogenic cells (Fig. 2 A5, B5, and C5).

### Histological detection revealed the endogenous origin and development of embryoids in FELBs

The frozen section technique was used to analyze the origin and development of FELBs. FELB embryoids of different sizes were sequentially formed in the translucent sticky callus, and FELBs were derived from cortex (root and stem explants) cells and mesophyll (leaf explant) cells (Fig. 3 A, B, C, D, and E). It also demonstrated that one individual FELB could contain many embryoids at different developmental stages (Fig. 3 F). The FELB embryoids could develop into spherical early globular embryos, which are dark spherical complexes, composed of embryogenic cells surrounded by non-embryogenic calli (Fig. 3 G). The individual FELBs could contain one or more embryoids, arranged in different forms, during the early developmental stage of SE (Fig. 3 H, I, J, and K). However, for late-stage SE, only globular- and torpedo- shaped embryos, and transitional types of heart and torpedo- shaped embryos (Fig. 3 L, M, N and O), were observed; cotyledonary- shaped embryos were not observed.

### Histological detection demonstrated the development of vascular tissue in FELBs and mature somatic embryos

The development of vascular tissue in FELB embryoids and somatic embryos developed from FELBs were investigated using the frozen section technique. It showed that vascular tissue developed with the development of embryoids in FELBs and somatic embryos (Fig. 4). The vascular tissues in FELB embryoids, and the mature embryos developed from FELBs, were separate from those of the parent explants (Fig. 4), confirming that the FELBs obtained could develop into real somatic embryos.

### Plantlets could develop from *in vitro* and *in vivo* FELBs

Two methods were used to induce plantlets from *in vitro* and *in vivo* FELBs. For plantlets developed from *in vitro* FELBs, FELBs separated from explants were transferred to the medium supplemented with 5.0 mg/L BAP and 0.1 mg/L GA_3_ for the development of regenerated plantlets. The frequency of regenerated plantlets developed from *in vitro* FELBs was 100.00% (Fig 5), and individual *in vitro* FELBs often developed into more than one plantlet (Fig 5. H), which is different from the usual scenario in other plant species in which the somatic embryo often forms individually and develops into one plantlet [35], [36]. For plantlets developed from *in vivo* FELBs, this study demonstrated that *in vivo* FELBs with parent callus transferred to a medium supplemented with TDZ could directly develop into plantlets, and supplemented with 20 mg/L TDZ resulted in a significantly higher frequency of the root, stem, and leaf explants than other concentrations (38.63%, 15.63%, and 61.30%, respectively; Table 3). In addition, individual *in vivo* FELBs (Fig 5. I-L) could develop into many multiple buds, and finally develop into many plantlets, as is the case for *in vitro* FELBs.

**Table 3:** Frequency of plantlet induction from *in vivo* frog egg-like bodies (FELBs) of *S*. *nigrum* root, stem, and leaf explants.

TDZ (mg/L)	Frequency of plantlets induction (%)		
	Roots	Stems	Leaves
0	0.00±0.00 hH	0.00±0.00 hH	0.00±0.00 hH
10	15.77±0.35 dD	4.53±0.27 eE	32.03±0.48 cC
20	38.63±0.41 bB	15.63±0.23 dD	61.30±0.48 aA
30	3.67±0.35 fEF	1.53±0.20 gG	3.07±0.30 fF

Note: Frequency of plantlet induction from *in vivo* FELBs of explants, as the number of induced FELBs as a proportion of the total number of explants inoculated. The mean and standard error per treatment were calculated from 300 explants from 30 petri dishes (as 30 replicates). Capital and lowercase letters represent significant differences at the 1% and 5% probability levels, respectively. Significant differences were analyzed by the Duncan test, using SPSS 10.0.

## Discussion

The optimization of supplementation with plant growth regulators is key to the success of SE and the regeneration of plants [37], [38]. In the present study, SE induction by FELBs in *S*. *nigrum* was achieved though supplementation with 2,4-D alone, without TDZ, whereas supplementation with NAA failed to induce FELBs. Light conditions are important for SE too; in this study, the explants being kept in the dark, instead of under moderate light as in previous studies [36], [39], resulted in successful FELB induction in *S*. *nigrum*. Supplementing with suitable 2,4-D, and cultivating in the dark, were the prerequisites for FELB induction in this species. Under the above conditions, SE by FELBs was successfully induced from the root, stem, and leaf explants of *S*. *nigrum* at a high frequency. Significant differences existed in FELB induction frequencies between the three explants (leaf 99.47%> root 86.97%> stem 83.30%). All the *in vitro* FELBs, despite the origin of their explants, could be induced into plantlets. This suggests that explants from different organs of *S*. *nigrum* could easily be induced into somatic embryos and further plantlets under optimal conditions, but with organ differences in SE induction ability.

In most indirect SE, loose white callus is first induced and individual somatic embryos develop on the callus. In this study, translucent sticky callus was first induced and FELB, a new SE structure in *S*. *nigrum*, also formed. Unlike in most SE, the induced callus was translucent and sticky, and multiple embryoids had been induced in the special callus. The translucent sticky callus could play a role in protecting and providing nutrients for the induced embryoids in it, like the structure of a frog egg cluster. SE by FELB can be considered a new pathway for the following reasons: 1) FELB is a new type of structure not reported before; 2) the induction conditions of SE by FELBs are different from those of a typical SE pathway; 3) individual FELBs can develop into multiple plantlets, whereas usually only one somatic embryo develops into one plantlet in a typical SE pathway; 4) The special callus formed at the early stages of SE by FELB is different from that in a typical SE pathway. However, further study is needed to ascertain whether FELBs are found in other plant species.

*S*. *nigrum* (nightshade) is a medicinal plant with therapeutic properties belonging to the *Solanum* genus, some accessions of which exhibit a high resistance to *Phytophthora infestans*, the causal agent of potato and tomato late blight [40]. Therefore, *S*. *nigrum* can not only be used as medical material, but also serve as a resource of disease resistance genes for the improvement of important *Solanum* crops, such as tomato and potato. The regeneration system developed by FELBs will benefit the establishment of transformation systems in *S*. *nigrum*, which will be helpful for the improvement of *S*. *nigrum*, and its relative *Solanum* crops, and clarify the mechanism of formation of FELBs in *S*. *nigrum*. The developed system can be used to obtain pathogen-free plants of *S*. *nigrum*, because the vascular tissue of somatic embryos is independent from the parental explants [41]. In addition, the plant regeneration system developed by FELBs may give clues to establishing similar systems in other *Solanum* crops, and even the crops recalcitrant in SE induction and regeneration.
