# Correction: Regeneration of *Solanum nigrum* by Somatic Embryogenesis, Involving Frog Egg-Like Body, a Novel Structure

**DOI:** 10.1371/journal.pone.0125645

**Published:** 2015-04-13

**Authors:** Kedong Xu, Yunxia Chang, Kun Liu, Feige Wang, Zhongyuan Liu, Ting Zhang, Tong Li, Yi Zhang, Fuli Zhang, Ju Zhang, Yan Wang, Wei Niu, Shuzhao Jia, Hengchang Xie, Guangxuan Tan, Chengwei Li

References 40 and 41 are incorrectly switched. The correct reference 40 is: Lebrecka R (2008) Host-pathogen interaction between *Phytophthora infestans* and *Solanum nigrum*, *S*. *villosum* and *S*. *scabrum*. European J of Plant Pathol 120(3): 233–240. doi: 10.5580/e27. The correct reference 41 is: Rohani ER, Ismanizan I, Noor NM (2012) Somatic embryogenesis of mangosteen. Plant Cell Tiss Org Cult 110(2): 251–259. doi: 10.1007/s11240-012-0147-4.
